# Hyperspectral and Color Imaging of Solvent Vapor Sorption Into Porous Silicon

**DOI:** 10.3389/fchem.2018.00610

**Published:** 2018-12-11

**Authors:** Soohyun Chun, Gordon M. Miskelly

**Affiliations:** School of Chemical Sciences, University of Auckland, Auckland, New Zealand

**Keywords:** porous silicon, hyperspectral imaging, sensor, vapor sensing, surface modification

## Abstract

A porous silicon thin film photonic crystal (rugate) sample with both a radial gradient in the rugate reflectance band wavelength and two spatially separated pore-wall surface chemistries (methylated and oxidized) was monitored by hyperspectral and color imaging while it was dosed with vapors of acetone, ethanol, heptane, 2-propanol, and toluene at concentrations ranging from 100 to 3,000 mg m^−3^. The shift in the wavelength of the rugate reflectance band maximum at each position along a transect across the two surface chemistries, as derived from the hyperspectral imaging, could discriminate between the different solvents and concentrations of solvents, while the change in hue derived from the color camera data along an analogous transect did not provide discrimination. The discrimination between solvents was mainly due to the two different surface chemistries, and the gradient associated with the change in the rugate reflectance band wavelength did not affect the selectivity significantly. There was spatial variability in the spectral and color responses along the transect independent of the overall rugate reflectance band wavelength gradient and pore-wall surface chemistries, and this was attributed to factors such as the presence of striations in the silicon wafer from which the porous silicon was prepared.

## Introduction

Porous silicon photonic crystal (rugate) substrates can act as optical transducers for the detection of volatile organic compounds (VOCs) that sorb or capillary condense within the pores. Most previous studies have monitored the response of porous silicon photonic crystal substrates to environmental conditions either via point measurements (e.g., Ruminski et al., 2010, 2011; Kelly et al., 2011b; Jalkanen et al., 2014) or using measurements integrated across an area of the porous silicon (e.g., Ariza-Avidad et al., 2014). An alternative approach is to individually monitor selected spatial areas across the substrate, and then combine those individual responses using multivariate analysis techniques. This approach can probe variability in the response due to deliberately imposed changes (e.g., surface chemistry or pore-size gradients) or other causes of heterogeneity (e.g., uncontrolled pore-size variations due to the substrate or experimental conditions).

Several recent papers have reported the use of porous silicon substrates with step changes in pore surface modification chemistry to obtain selectivity in response toward analytes which penetrate the pores. Thus, Wu et al. (2013) prepared uniformly-etched porous silicon and then used a masking technique to prepare sensors that were methylated on one half and oxidized on the other. The response of these sensors to solution analytes of differing polarity were then examined by monitoring the visible reflectance spectrum of a small disc-shaped region of the substrate that included similar proportions of the two surface chemistries. A similar experimental design was reported by Murguía et al. (2013), except that they used a sinusoidally varying current when preparing their porous silicon thin film, to prepare one-dimensional photonic crystals (rugate porous silicon). These authors monitored a small region that included their two surface chemistries (oxidized silicon and hydrosilylated silicon), and then analyzed the temporal data using wavelets. In a third example, Sweetman and Voelcker (2012) used photoresist patterning to prepare porous silicon films with different surface chemistries, and then monitored a region of the porous silicon that included the two surface chemistries (modified with pentafluorophenylsilyl and aminopropylsilyl groups). In this case the hydrophobic and hydrophilic nature of the surface modification was designed so that the two analytes (toluene and ethanol) were preferentially sorbed by the hydrophobic layer while the hydrophilic layer provided a fiducial spectral marker.

The stability and relative response toward organic vapors of porous silicon sensors with different surface modifications was described by Ruminski et al. (2010). Of relevance to the current study, they noted that thermally-oxidized silicon and methylated silicon had good stability in ambient air, and that the oxidized surface responded strongly to isopropanol, with a shift in the wavelength of the rugate reflectance band of over 10 nm. In contrast, exposure of the oxidized porous silicon to heptane resulted in a <2 nm shift, while exposure of 2-propanol or heptane to the methylated porous silicon caused very similar shifts, both <2 nm.

An example of imaging porous silicon where the response signal was determined by integrating over a large region of the porous silicon was a study by Ariza-Avidad et al. (2014) on the degradation of porous silicon samples when immersed in aqueous solutions. This study reported that the hue of the samples had a non-monotonic behavior during the degradation process and therefore preprocessing was performed to extract a “hue-like parameter” that changed in a monotonic way with the sample degradation.

Hyperspectral imaging of porous silicon has been conducted by Leacock-Johnson et al. (2013), who prepared porous silicon with a pore wall surface chemistry gradient from hydrophobic (methyl terminated) to hydrophilic (pentyl alcohol terminated) and then monitored the spectral response as ethanol was titrated into water covering the porous silicon. This study demonstrated that there was an overall monotonic change in the extent to which the alcohol-water solution penetrated the porous silicon along the transect, but some of the data reported in that paper also show small deviations from the overall trend along the transect that could be ascribed to factors such as the presence of striations in the initial silicon used to form the porous silicon. The solution infiltration experiment caused large spectral changes, with the wavelength of the rugate reflectance maximum moving 30–50 nm. This change is sufficiently large that smaller scale variations due to factors such as striations did not cause significant effects on plots of the observed wavelength maxima vs. time. Steady state hyperspectral imaging of porous silicon was also reported by Miskelly (2016).

The current study extends the above examples. The porous silicon was prepared using a temporally sinusoidal etching current so that it has a prominent rugate reflectance band (Lorenzo et al., 2005), but also has measurable interference fringes to the red (longer wavelengths) of the rugate peak. Furthermore, preparation of the porous silicon used a spatially varying current density so that there is a radial gradient in wavelength of the rugate reflectance band across the porous silicon. Two pore-wall surface modifications were performed on adjacent sections of the porous silicon, using the strategy reported by Wu et al. (2013). The change in the wavelength of the rugate reflectance band or in the hue was then monitored as the sample was exposed to organic solvent vapors. This resulted in much smaller wavelength or hue shifts than were observed in the Leacock-Johnson et al. (2013) solution immersion study, which meant that drift and porous silicon inhomogeneities had a relatively greater impact on the data than was reported by those authors. A comparison of data collected under almost identical conditions with a line-scan hyperspectral imager (Sigernes et al., 2000) and with a color camera allowed the comparison of the sensitivity of measurements using the two imaging modes.

## Materials and Methods

A silicon wafer (Siltronix, 100 single side polished B doped, 0.8–1.2 mΩ-cm, thickness 500–550 μm) was cut into approximately 2 × 2 cm pieces, which were immersed in 2-propanol (LR) and placed in an ultrasonic bath for 10 min. The samples were then dried using nitrogen and mounted into a Teflon etch cell using procedures based on those of Sailor (2012). All electrochemical procedures used a PAR EG&G Model 173 potentiostat/galvanostat. Where necessary, sinusoidal waveforms created using Tektronix ArbExpress AXW100 version 2.0.2005.30 software were input to a Tektronix AFG3021 single channel Arbitrary/Function generator, with the output applied to the PAR 173 input. A pre-etch was performed using 3:1 (v/v) 48% HF: ethanol and a platinum (Aldrich, 99.99%) ring counter electrode. A current density of 46.9 mA cm^−2^ was applied for 30 s with the silicon as the anode to create a thin sacrificial porous layer. The silicon was then rinsed with ethanol three times and the thin sacrificial porous layer was removed by treatment with 1 M aqueous KOH containing 10% (v/v) ethanol for 2 min. The sample was then rinsed with ethanol two times.

The porous silicon samples were prepared using electrochemical etching with a straight Pt wire counter electrode (Aldrich, 99.99%), placed perpendicularly to the silicon anode so that the wire end was about 2 mm above the center of the silicon. Etching was performed using 3:1 (v/v) 48% HF: ethanol solution. Etching used a sinusoidal waveform of 40 cycles in which the current density varied between 35.2 and 48.9 mA cm^−2^ and was applied for 750 s. After the etching, the sample was rinsed with absolute ethanol two to three times, and dried using nitrogen while still within the etch cell.

Two pore-wall surface chemistries were imposed on a single sample of porous silicon using a recently reported masking method (Wu et al., 2013). First, a KSL-1100X-S compact muffle furnace (MTI Corporation) with a programmable controller was used to partially thermally oxidize the porous silicon. The furnace temperature was ramped to 300°C over 60 min at 5°C min^−1^ then the temperature was held at 300°C for 30 min before the furnace was allowed to cool to room temperature. The porous silicon sample was placed inside a small open crucible and was in the furnace for the complete thermal cycle. A small drop of 13% (w/w) polystyrene (M_W_~ 280,000) dissolved in toluene was then painted onto half of the porous silicon sample using a small flat paint brush. The sample was then immersed in HF:ethanol (1:1 v/v) for 2 min to dissolve the exposed oxidized porous silicon and then rinsed with ethanol prior to selective chemistry being performed on the unmasked side as described below.

The masked porous silicon sample was mounted into the same electrochemical cell as was used for porous silicon etching, with an adapter that allowed white light irradiation of the porous silicon during electrochemistry under an inert atmosphere. The solution used for electrochemical methylation was prepared by transferring 0.27 g of lithium iodide, 10 mL of anhydrous acetonitrile, and 0.44 mL of iodomethane into a round bottom flask and then freeze-pump-thawing the solution three times. The electrochemical cell was evacuated then filled with nitrogen three times before the electrochemical methylation solution was introduced under nitrogen. A current density of 10.16 mA cm^−2^ was applied with the porous silicon as the cathode for 1 min, while the porous silicon was illuminated through the glass adapter with white light from a Rofin PL-10 Polilight. The electrochemical cell was then disassembled and the porous silicon was immediately acidified using glacial acetic acid. The porous silicon was then rinsed three times with glacial acetic acid, three times with acetonitrile, and then three times with ethanol. The electrochemical cell was then disassembled and the sample was immersed in a solution of pentane before air drying. Finally, the polystyrene masking was removed by immersing the sample in toluene for at least 30 min. The sample was then rinsed three times with toluene, followed by ethanol. The sample was then immersed in a solution of pentane before drying.

A Philips XL30S Field Emission Gun Scanning Electron Microscope (FEG SEM) with a SiLi Super Ultra-Thin Window detector was used to obtain cross sectional and surface images of the fabricated porous silicon samples. The SEM images were taken using the high resolution and ultra-high resolution modes operating at electron accelerating voltages of 5 kV in a vacuum.

A Perkin Elmer Spectrum Two 100 FT-IR Spectrometer with attenuated total reflectance (ATR) attachment was used to collect Fourier transform infrared spectroscopy (FT-IR) absorbance spectra. Perkin Elmer spectrum 10^TM^ software was used to collect and anayse the spectra, which were averages of 32 scans with a resolution of 4 cm^−1^ over the range 600 to 4,000 cm^−1^. The background was collected with the ATR crystal exposed to air.

A KSV Cam 100 tensiometer with Attension Theta analysis software was used to determine the water contact angles for the porous silicon samples. Measurements were performed three times for each different surface, and the average water contact angle is reported with calculated standard deviation.

A scientific charge coupled detector camera (QICAM, Q-Imaging) with Varispec Liquid Crystal Tunable Filter (LCTF) (CRI Ltd) was used to obtain a hyperspectral image cube of the complete surface of a modified porous silicon sample from 450 to 720 nm at 5 nm intervals (55 bands). In- house code in the V^++^ program (version 5.0.0.301 Digital Optics Ltd ®) was used to control image collection. An image cube of a specular reflectance standard was also collected to allow determination of reflectance. The saved images were averages of 4 images with a constant shutter time of 30 ms. A Fiber-Light DC-950 illuminator (Dolan Jenner) with a Fiber-Lite Diffuse Axial Illuminator (Edmund Scientific Optics) was used as the light source. The collected images were processed using the Matlab software® R2014a (The MathWorks, Inc.). The image cubes for the sample, reference standard, and dark image were first imported and cropped to isolate the porous silicon. The same cropping coordinates were used for the reference standard and dark images. One dimension of the cropped image cube corresponds to a spectrum at each image pixel, and these spectra were processed to determine the wavelength of the rugate band at each position of the hyperspectral image using a modified version of the Matlab function findpeaks.m, (O'Haver, 2018).

Two different imaging systems were used to image the porous silicon samples during vapor dosing. A Point Gray Research® Flea 2G 13S2C-C model camera (F2G) with a 50 mm double Gauss lens (Edmund Scientific Optics) was used for color (RGB) imaging. A line-scan hyperspectral imager constructed with a Point Gray Research® Flea 2-03S2M grayscale camera and a 50 mm double Gauss imaging lens was used for hyperspectral imaging during vapor dosing, and this system was calibrated using an HG-1 mercury-argon lamp (Ocean Optics, Inc.). The porous silicon was mounted in the vapor dosing cell so that the porous silicon surface was perpendicular to the selected imaging system. The position of the vapor dosing cell was arranged to provide maximum reflectance for the hyperspectral imaging, and so that the porous silicon filled the field of view for the RGB imaging. The vapor dosing cell was constructed of Teflon, with an A48-927 anti–reflection coated glass slide (Edmund Optics) as the window.

An automated vapor dosing system was used to generate pulses of vapor at known concentrations for known periods of time. The vapor dosing system used in this research was designed and developed in-house at the University of Auckland and characterized by Wong (2012). Vapors of five solvents: acetone, ethanol, heptane, 2-propanol, and toluene were generated using the automated vapor dosing system. The concentrations used were 100, 300, 700, 1,000, 1,500, and 2,000 mg m^−3^ for acetone and 2-propanol, 100, 300, 700, 900, 1,100, and 1,500 mg m^−3^ for ethanol, 200, 400, 800, 1,500, 2,000, and 2,500 mg m^−3^ for heptane, and 200, 400, 800, 1,600, 2,000, 2,500, and 3,000 mg m^−3^ for toluene. A pre-dose of the system with 1,000 mg m^−3^ of the next solvent followed by a purge period was performed prior to measurenment with each solvent. The purge periods during dosing for hyperspectral imaging was 20 min while the dose period was 30 min. The purge period for RGB imaging was 40 min with dosing for 30 min. A Baseline®-MOCON® PID-TECH® plus photoionization detector was connected to the outlet of the vapor dosing cell to independently monitor the solvent vapor concentration. TRAQ-WARE software recorded the concentration (ppm) of the vapor every 2 s.

The hyperspectral images were collected every 30 s during the dosing cycle for each solvent, with ten images being averaged at each time point. Images for each dosing experiment were saved directly to a computer, and were analyzed off-line using Matlab. The region of interest of each image corresponding to the porous silicon was selected, resulting in cropped images of 221 by 370 pixels. The hyperspectral images were corrected for the estimated spectral envelope of the system response, using the obtained raw image cube data. First, the minimum value of the image cube was subtracted from all the other values. The column means of the resulting matrix were calculated, and then the row means of these column means were calculated. This calculation resulted in a vector of average brightness with respect to wavelength. This vector of average brightness was then normalized and replicated to create a hyperspectral cube that contained the normalized mean spectrum for every pixel position and every time point. The raw image data was then divided by this mean spectrum, and this corrected data matrix was either used directly in subsequent multivariate data analyses or the wavelength of the rugate reflectance maximum in each spectrum was extracted, and these wavelengths were used in the multivariate analyses.

Color (RGB) images of the porous silicon during vapor dosing were collected by averaging 10 RGB images every 60 s and saving the average images directly to a computer. The images were imported into Matlab, and a 801 by 21 pixel region of interest corresponding to a transect down the center of each porous silicon image was isolated by cropping. The row means of each of the red, green, and blue channels for this transect were calculated and then used to calculate the hue for each transect position, using the following equation

$$\begin{array}{l} hue=cos^{-1}\left[\frac{0.5\times \left(red-green\right)+\left(red-blue\right)}{{\left[{\left(red-green\right)}^{2}+\left(red-blue\right)\left(green-blue\right)\right]}^{1/2}}\right] \end{array}$$

The difference in hue at a given point and time compared to the initial hue at that point was then calculated. The RGB hue difference data required spatial filtering to improve the signal to noise ratio of the processed data. This was performed using the 3 X 3 median filtering function of Matlab “medfilt2.” Following this preprocessing the data were analyzed using principal component analysis and linear discriminant analysis in Matlab.

## Results

### Preparation and Characterization

The porous silicon was prepared using a sinusoidal etching current density and with a thin Pt wire counter electrode placed perpendicularly above the center of the silicon wafer, so that the resulting porous silicon was a rugate filter (photonic crystal) with a radial gradient wavelength of the rugate reflection band (Li et al., 2005). The average pore size in such porous silicon structures has also been shown to vary with current density (Collins et al., 2002; Clements et al., 2012) so that there should also be a radial gradient in pore size. The porous silicon was then modified with two different pore-wall surface chemistries as described by Wu et al. (2013). The porous silicon was partially thermally oxidized, and then half of the porous silicon was impregnated with polystyrene. The oxidized porous silicon was removed from the non-protected half using dilute HF solution, and then the exposed hydrogen-terminated porous silicon was immediately methylated via the electrochemical reduction of iodomethane with the porous silicon as the cathode (Gurtner et al., 1999). Finally, the polystyrene was dissolved to expose the remaining oxidized porous silicon. This procedure gave porous silicon samples that had an oxidized (hydrophilic) portion and a methylated (hydrophobic) portion and also had a radial gradient in pore-size. These are referred to as OM samples in the following text.

**Figure 1:**
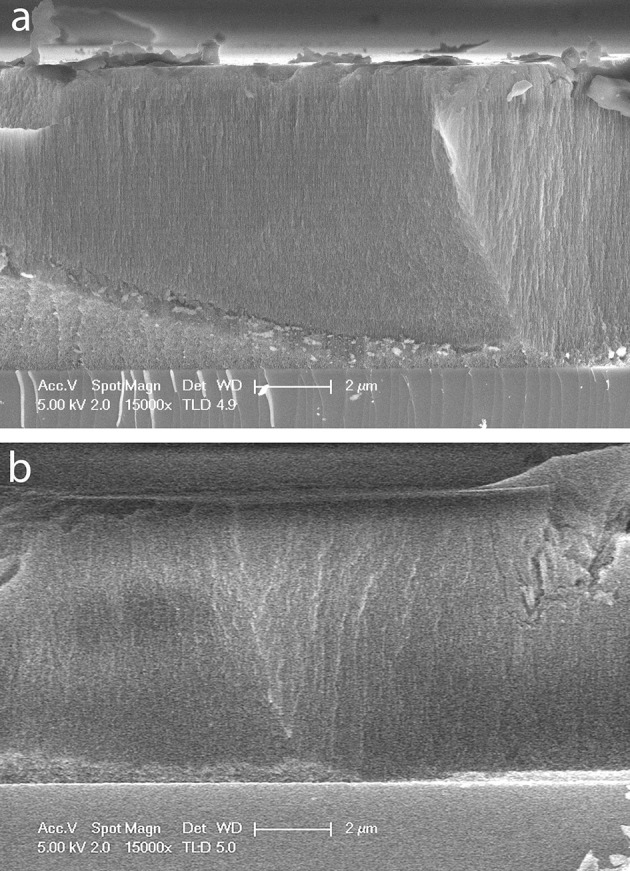
SEM images of cross sections of a sample of OM porous silicon. **(a)** Oxidized side of porous silicon **(b)** methylated side of porous silicon. In each image the scale bar is 2 μm.

A cross-sectional SEM image of a small region of the oxidized portion of an OM porous silicon sample prepared as described above is shown in Figure 1a. The thickness of 8.7 ± 0.1 μm appears constant across the image with faint horizontal banding due to the periodic change in porosity caused by the rugate filter formation. A cross-sectional SEM image of part of the methylated portion of sample OM is shown in Figure 1b. The calculated average thickness of the methylated side (8.1 ± 0.1 μm) is slightly smaller than that of the oxidized side at a similar position along the radial gradient.

**Figure 2:**
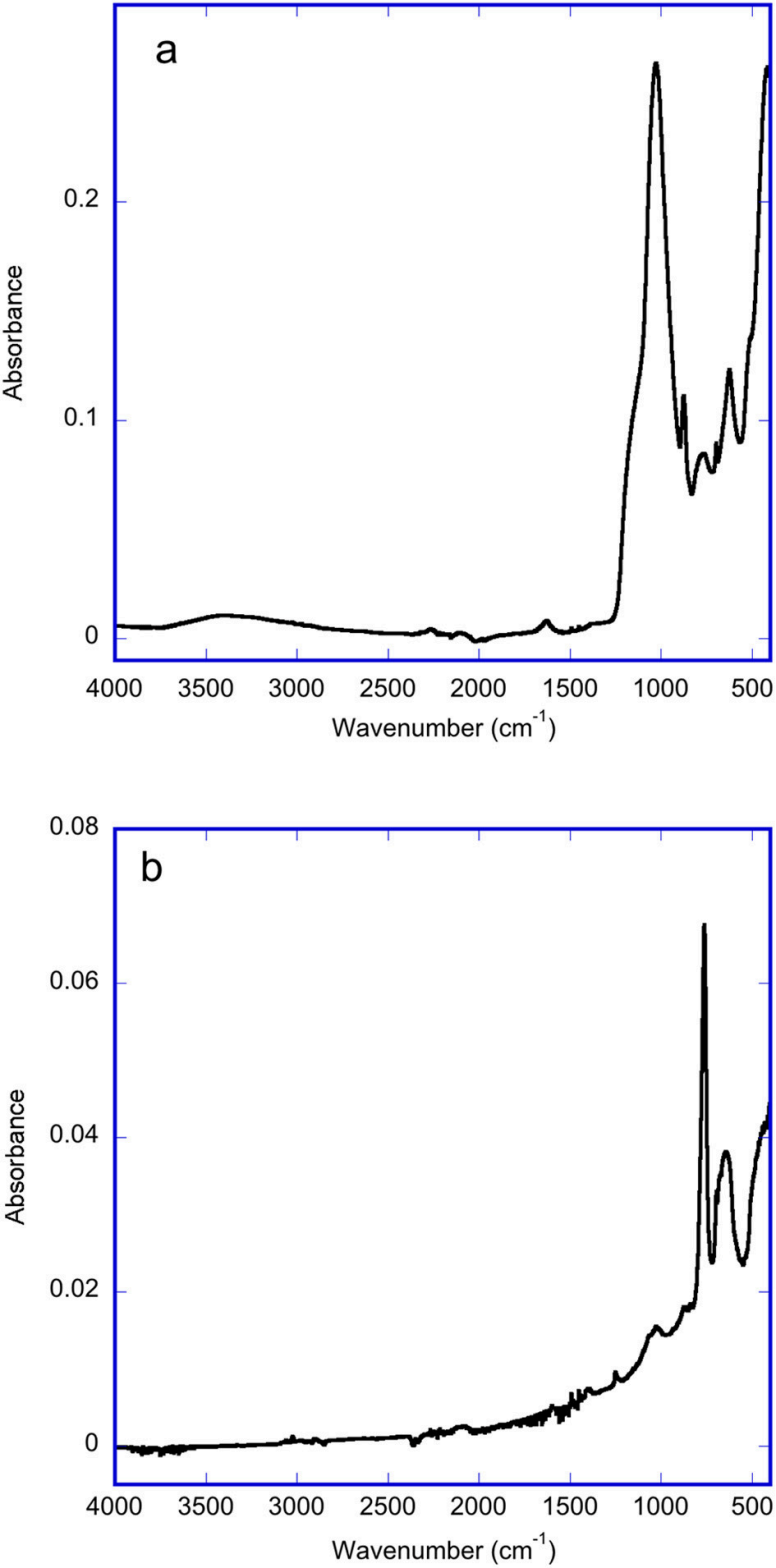
ATR-FTIR spectra from portions of each side on a sample of OM porous silicon. **(a)** Oxidized side of porous silicon, showing Si-O stretching band at 1,038 cm^−1^ **(b)** Methylated side of porous silicon showing CH_3_ rocking band at 763 cm^−1^.

The ATR-FTIR spectrum of the oxidized portion of OM porous silicon (Figure 2a) shows a large Si-O stretching band with a maximum at 1,038 cm^−1^. The methylated portion of OM porous silicon should mainly be CH_3_ terminated, and the ATR-FTIR spectrum shows a sharp rocking band of the methyl groups at 763 cm^−1^ with two weak bands at 1,403 and 1,252 cm^−1^, which are associated with -CH_x_ deformation modes (Figure 2b). The spectral position of the rocking band is similar to that reported earlier (Canaria et al., 2002). The difference in the pore wall surface chemistry for the two halves of OM porous silicon is also shown by water contact angles. The oxidized portion gave a water contact angle of 70.8 ± 1.4° (*n* = 3) while the methylated portion had a contact angle of 131.2 ± 0.8° (*n* = 3).

**Figure 3:**
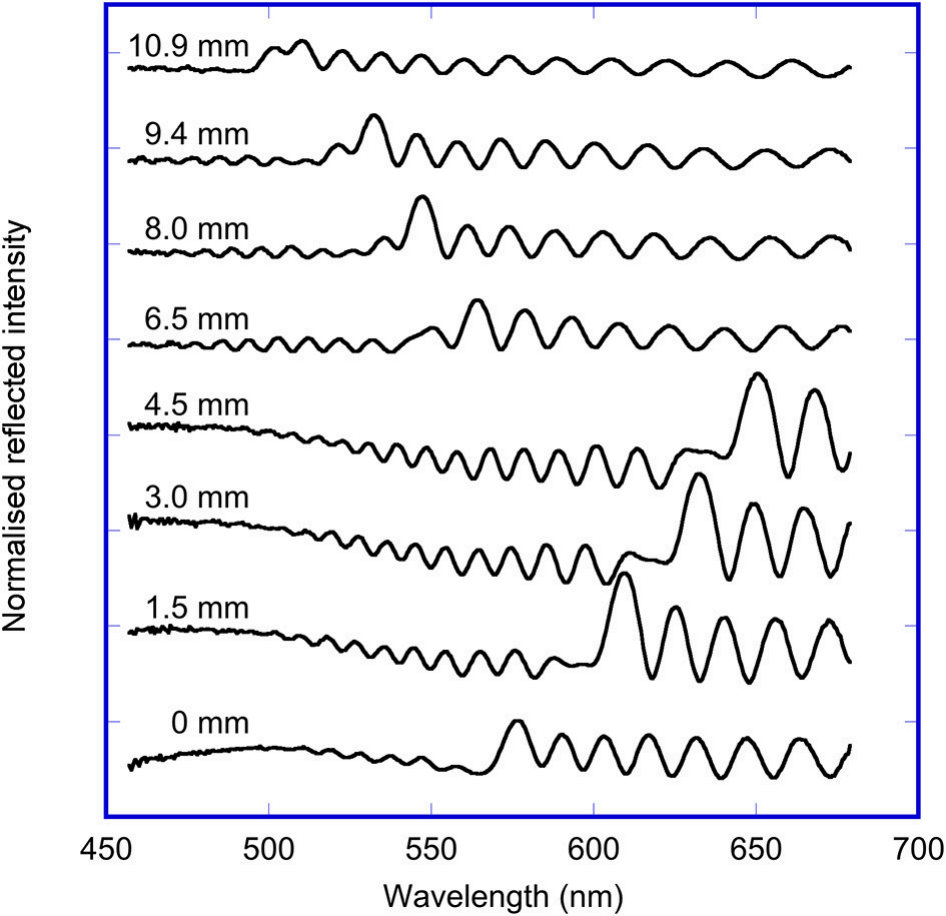
Reflected light spectra measured at specified points along a transect across the OM sample, starting at the oxidized side (0–4.5 mm) and ending on the methylated side (6.5–10.9 mm).

The UV-Visible reflectance spectra at selected points along a transect of the OM porous silicon sample are shown in Figure 3. The wavelength of the maximum of the rugate reflectance band on the oxidized side varied from 550 to 620 nm, while the wavelength of the maximum of the rugate reflectance band on the methylated side varied from 500 to 550 nm. These differences in wavelength on the two sides are due to the fabrication process of the sample. The removal of pore wall material prior to the methylation of the porous silicon has resulted in a blue shift of the rugate band. The magnitude of the rugate reflectance band for this OM sample were not much larger than the heights of the thin film interference fringes also observed in the UV-Vis spectra. However, the rugate peak can be identified as the significant peak with the shortest wavelength.

**Figure 4:**
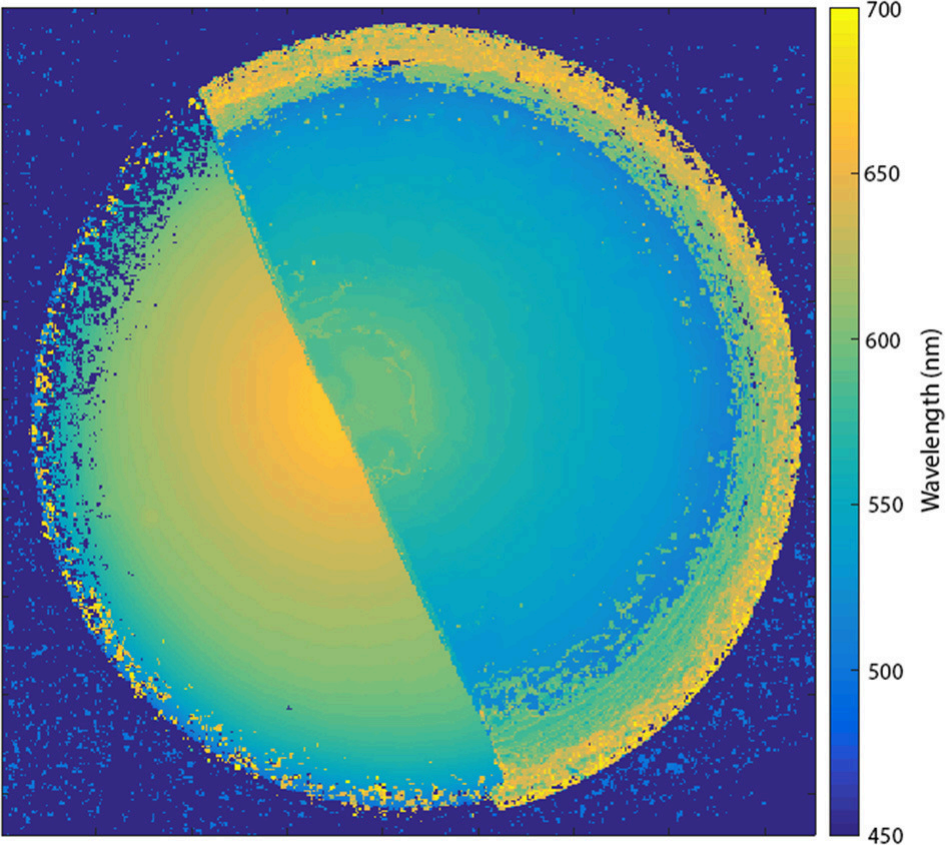
False color map showing the wavelength of the maximum of the rugate reflectance band at all positions of the porous silicon. The wavelengths were extracted from a hyperspectral cube collected with a Varispec LCTF and camera.

A false color map showing the wavelength of the maximum in the rugate reflectance band at all positions of an OM porous silicon sample is shown in Figure 4. The wavelength of the rugate peak maximum had approximately radial symmetry across each half of the porous silicon sample, with the longest wavelength near the center. The magnitude of the reflectance between the two surfaces were different from each other, and so the transition line between the surfaces could be distinguishable in single waveband images and the color difference could be observed by eye.

### Vapor Dosing Results

The optical responses of the porous silicon samples during exposure to solvent vapors were monitored using hyperspectral and RGB imaging techniques. Hyperspectral images were collected using a custom built line scan imager (Sigernes et al., 2000; Leacock-Johnson et al., 2013), while the RGB images were acquired with a scientific color camera. The wavelength and spatial calibration of the hyperspectral imager were performed using an atomic line calibration light source and a precision ruler, respectively. The hyperspectral images were processed to determine the wavelength of the maximum of the rugate reflectance band at each point along the transect, and changes in these wavelengths were used to monitor the exposure of the porous silicon to organic solvent vapors.

A slight drift was sometimes seen in the rugate peak wavelength values (for the hyperspectral image data) and the values of the hue (for the RGB image data) during a given vapor dosing experiment, and different experiments showed different patterns and magnitudes of the drift. The drift on the two sides could differ. For example, during 2-propanol dosing the wavelength of the maximum in the rugate reflectance band on the oxidized side decreased by up to 1 nm while on the methylated side it increased by up to 0.5 nm. All the drifts for the hyperspectral data were <1 nm, however this change was sometimes significant compared to the observed changes resulting from a vapor concentration change. Thus, the largest change in the wavelength of the rugate reflectance band due to dosing for the methylated side of sample OM during dosing of 2-propanol vapor was about 0.5 nm. It was therefore important to correct this background drift prior to any multivariate analysis. Separate monitoring of the temperature next to the porous silicon indicated that the drift was not caused by the small changes in temperature (up to 1°C) of the sample during dosing experiments. Although the actual cause of the drift was not determined, one possibility is that it was due to a slight change in the intensity or temperature of the light source, which could affect the observed wavelength of the rugate peak. Baseline correction was performed using the Matlab function bf.m applied to time points during the known purge periods. Since the dose and purge timing for the dosing experiments was always the same this background correction could be readily implemented. For most baseline corrections two points of the purge period were selected; one timepoint early in the purge period, and one timepoint near the end of the purge period. For 2-propanol, only one value toward the end of the purge period was selected to define the background, due to the slow desorption of 2-propanol from the oxidized porous silicon during the purge periods.

The data matrices containing the changes in the rugate peak wavelength (for hyperspectral image data) and median filtered change in hue (for RGB and Mode 0 image data) during vapor dosing with five different solvents were represented as false color maps, where the vertical axis is the distance along the transect, the horizontal axis is time, and the color represents either the change in wavelength or the change in hue at a given position compared to the initial value. The upper portion of each false color map represented the oxidized side of the porous silicon and the lower portion represented the methylated side. Depending on the polarity of the vapor and the polarity of the porous silicon surface, the magnitude of the response to a given solvent and concentration were different for each side of the porous silicon. Solvents with higher polarity showed higher responses on the oxidized (hydrophilic) side than on the methylated (hydrophobic) side.

**Figure 5:**
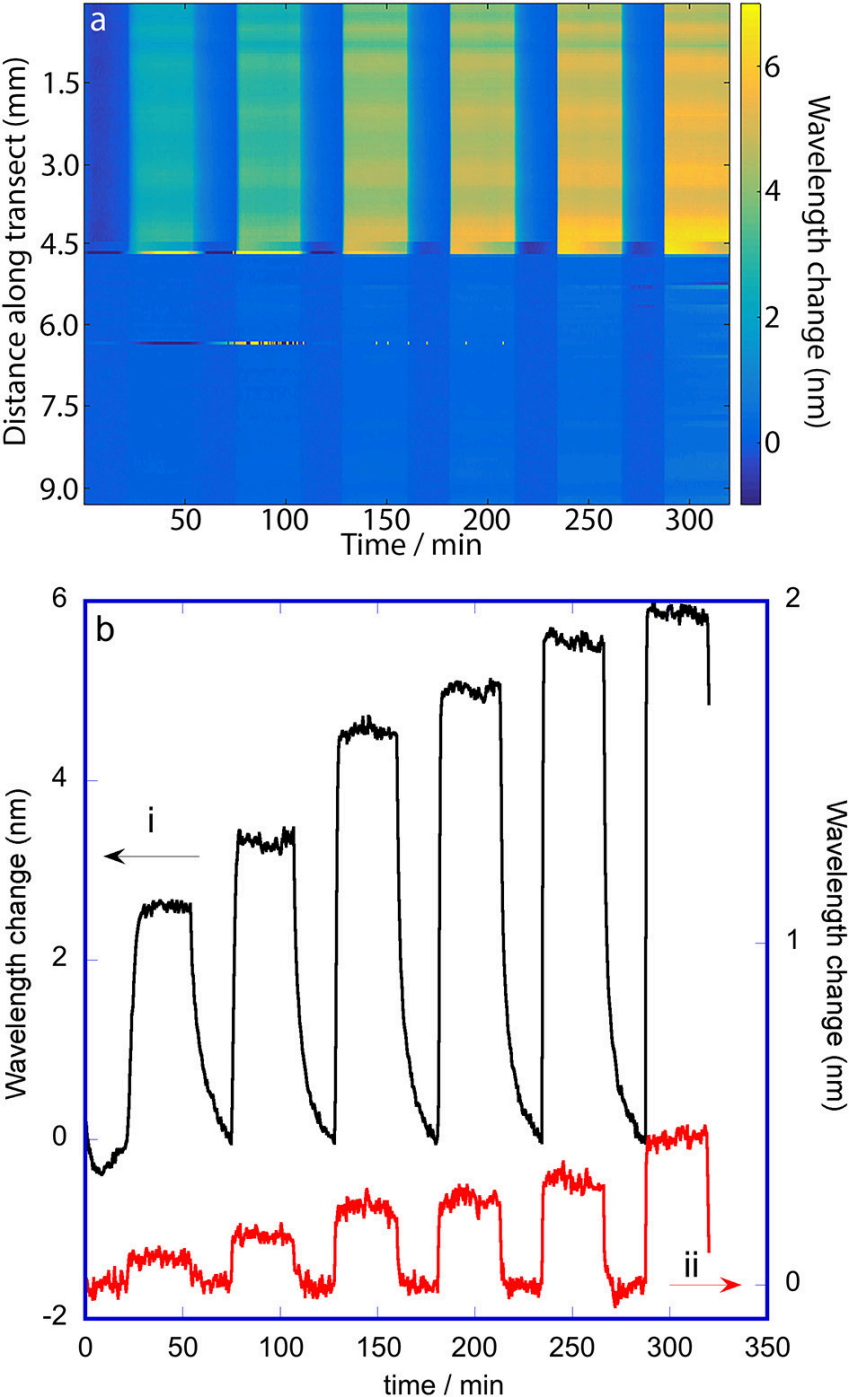
Change in wavelength of the rugate reflectance band maximum during dosing of the porous silicon with 2-propanol. The wavelengths were extracted from a hyperspectral cube collected along a transect across the OM porous silicon with a line-scan hyperspectral imager. **(a)** False color map showing changes in wavelength of the rugate reflectance band as a function of distance along the transect and with time. The vertical bands correspond to dosing and purge periods; the 2-propanol doses were 100, 300, 700, 1,000, 1,500, and 2,000 mg m^−3^. **(b)** Change in wavelength of the rugate reflectance band at two single positions along the transect: 4 mm (oxidized side) and 8 mm (methylated side).

**Figure 6:**
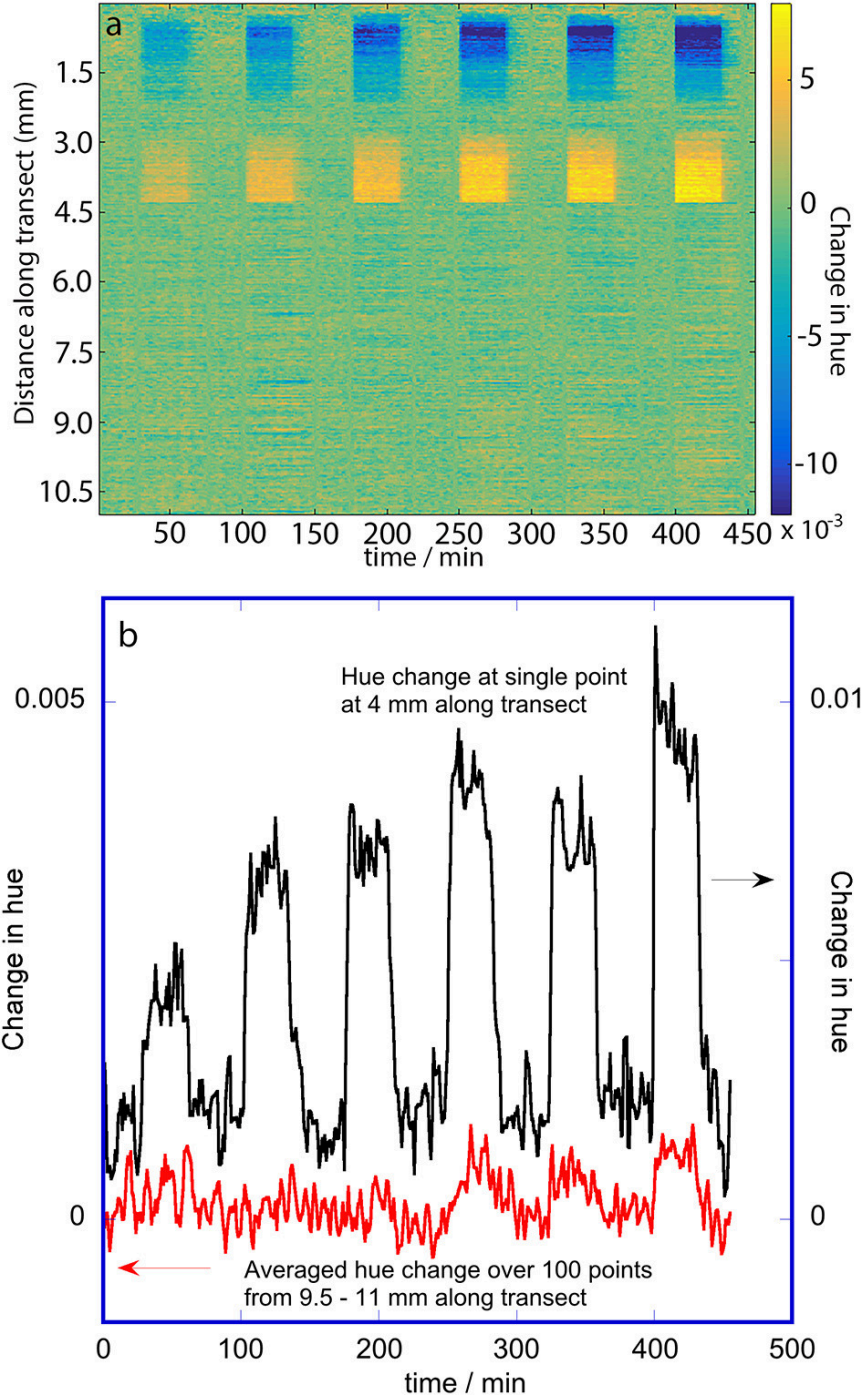
Change in hue of the porous silicon during dosing with 2-propanol. The hues were extracted from RGB (color) images. **(a)** False color map showing changes in hue as a function of distance along the transect and with time. The vertical bands correspond to dosing and purge periods; the 2-propanol doses were 100, 300, 700, 1,000, 1,500, and 2,000 mg m^−3^. **(b)** Change in hue at two selected places along the transect: 4 mm (oxidized side; single position) and 9.5–11 mm (methylated side; 100 positions averaged).

The false color maps generated from hyperspectral and RGB imaging during 2-propanol vapor dosing are shown in Figures 5a, 6a, respectively. 2-Propanol was dosed in the order; 100, 300, 700, 1,000, 1,500, and 2,000 mg m^−3^, so 13 vertical bands (7 purge bands, and 6 dose bands) can be seen in the false color map for RGB imaging. The false color map for the hyperspectral imaging only shows 12 bands because the image collection was stopped near the start of the final purge. The false color map for the hyperspectral imaging (Figure 5a) and the plot of wavelength changes at selected pixels in Figure 5b shows that the shift in wavelength of the rugate peak during vapor dosing of 2-propanol on the oxidized side is larger than on the methylated side. The change is the greatest during the final dose period, which is when the concentration of the 2-propanol vapor is the highest. The first (lowest) dose resulted in a rugate peak wavelength shift of about 2.5 nm on the oxidized side, while the last (highest) dose caused a rugate wavelength shift of about 6 nm. The wavelength shift on the methylated side of the porous silicon is much smaller than for the oxidized side with the change being around 0.1 to 0.5 nm over the vapor dosing experiment. The transition between the two pore wall surface chemistries can be clearly observed by this difference in response. This result shows that 2-propanol is sorbed by the oxidized (hydrophilic) side more than the methylated (hydrophobic) side. On the oxidized side of the OM porous silicon, additional variation in the response appearing as horizontal bands across the color map in Figure 5a are due to differences in pore sizes or other pore properties at different points across the porous silicon surface. The plots in Figure 5 also show tailing of the response on the oxidized side after the higher concentration dosing periods, as the 2-propanol slowly desorbs.

The false color map of the change in hue for the RGB image data during 2-propanol dosing (Figure 6a) shows that some portions of the oxidized side are responsive with small changes in hue while the methylated side shows very slight evidence of vertical bands corresponding to very small changes in hue. The oxidized side has a change in hue near the edge of the porous silicon that is opposite in direction to the change in hue near the transition line between the two pore wall surface chemistries. This non-monotonic behavior of hue with respect to change in the rugate wavelength was also noted by Ariza-Avidad et al. (2014). Since the change in hue on the oxidized side is greater than on the methylated side, the transition between the two pore wall surface chemistries can again be seen. The small hue change resulted in a much smaller signal to noise ratio compared to the wavelength changes determined with the hyperspectral imager. Thus, the plot of the response at a single position on the oxidized half of the porous silicon (Figure 6b) is much noisier that the similar plot in Figure 5b, and the hue change at 100 positions on the methylated side were averaged to obtain the smaller plot in Figure 6b, which still has a poor signal to noise ratio.

Dosing with the other solvent vapors led to similar false color maps, with decreased responses at the same concentration. Dosing with heptane led to the smallest responses, and the responses to this solvent on the oxidized and methylated sides of the porous silicon were very similar.

### Multivariate Analysis of the Images of Sample OM During Vapor Dosing

**Figure 7:**
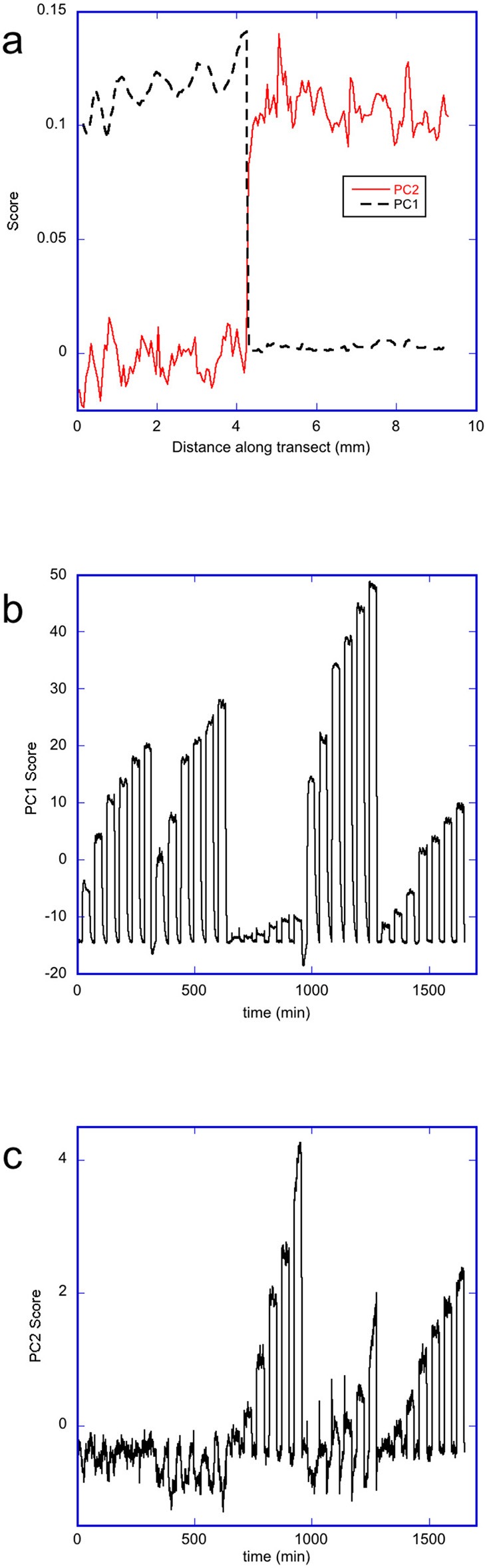
Principal component analysis of the changes in wavelength of the rugate reflectance band as the porous silicon OM sample was dosed with 5 different solvents in the order acetone, ethanol, heptane, 2-propanol, and toluene. **(a)** Plot showing loadings of PC1 and PC2 along a transect across the OM porous silicon **(b)** Plot showing the scores for PC1 with time during dosing **(c)** Plot showing the scores for PC2 with time during dosing.

The data matrices of the changes in the rugate peak wavelength upon dosing with each solvent were concatenated into a combined data matrix. Each row of the individual matrices matched in spatial position, and the combined matrix contained all the dosing pulses (as a function of time) in the order: acetone, ethanol, heptane, 2-propanol and toluene. Principal component analysis was performed on the transposed combined data matrix. The first three principal components explained 98.9, 0.78, and 0.08% of the variance in the data respectively. Plots of the scores and loadings for the first two principal components (labeled PC1 and PC2 respectively) are shown in Figure 7. The PC loadings, Figure 7a, are related to the contribution of changes in the wavelength of the rugate reflectance band at each position along transect to each principal component. The abrupt change in plots of the PC1 and PC2 loadings vs. pixel position is due to transition between the two pore-wall surface modifications, where the first part corresponds to the oxidized side, and the second part corresponds to the methylated side. The loadings of PC1 are higher for the oxidized side than the methylated side, while the loadings for PC2 are higher for the methylated side than the oxidized side. The PC3 plot loadings showed little difference in average magnitude between the two sides, however the values on the oxidized varied more than on the methylated side. The plots of the PC1 and PC2 scores for the change in the rugate peak wavelength during dosing are shown in Figures 7b,c. The PC1 score plot shows pulses due to the dosing of acetone, ethanol, heptane, 2-propanol and then toluene. The first six pulses represent acetone at six concentrations, the next six pulses represent ethanol at six concentrations and so on. The PC1 scores for 2-propanol are the highest, and then ethanol, acetone, toluene, with heptane showing the lowest PC1 scores. The PC2 scores also show a set of pulses with time, but heptane shows the greatest values followed by toluene and propan-2-ol, with ethanol being only slightly higher than acetone. Most of the scores for principal component 3 (PC3) were near zero, with some large spikes which corresponded to when the system was transitioning between purge and dose periods.

Apart from the two pore-wall surface chemistries, the porous silicon also had a radial gradient in pore sizes. The PC1 loadings show a very small magnitude trend which may correspond to this radial gradient on the oxidized side underlying the more obvious short-scale variability. The PC3 loadings also showed longer- scale as well as shorter-scale changes in response over the oxidized side. From Figure 7 it appears that small-scale variations (e.g., due to the striations which originate from different dopant densities in the silica substrate (Jastrzebski et al., 1980; Fusegawa and Yamagishi, 1992; Schweizer et al., 2010), dominate over effects due to the radial porosity gradient.

**Figure 8:**
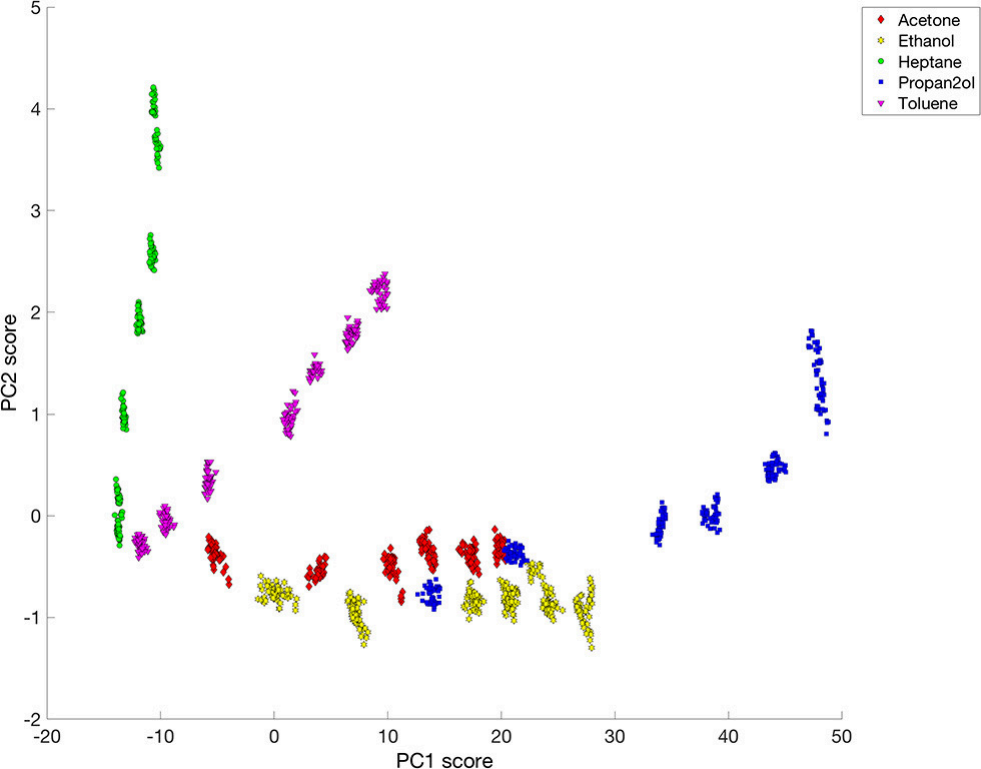
Plot of PC2 vs. PC1 for rugate reflectance band wavelength shift data corresponding to steady-state dosing or purging of acetone, ethanol, heptane, 2-propanol and toluene.

The initial principal component analysis had significant contributions from changes occurring during the transitions from purge to dosing and from dosing to purge, particularly for PC3. In order to avoid the responses during the transition periods dominating or perturbing the analysis, the PCA was repeated using just selections of 40 data points obtained during the steady-state phase of each solvent dosing pulse at each pixel position. A plot of the PC2 scores vs. the PC1 scores from this analysis, Figure 8, shows clear separation of heptane, toluene and the other three solvents–acetone, ethanol, and 2-propanol. The most hydrophobic solvent, heptane, has lower PC1 scores but large PC2 scores, while the opposite is true for the more hydrophilic vapors (acetone, ethanol, and 2-propanol). Toluene is more hydrophilic than heptane, but is also more hydrophobic than acetone, ethanol and 2-propanol, so the score values of toluene are located between the data corresponding to heptane and the other three solvents. For each solvent, all concentrations could be distinguished. However, acetone, ethanol and 2-propanol all lie on similar curves. A similar plot of PC3 scores against PC1 scores for the change in rugate peak wavelength upon dosing with all the solvents (not shown) showed some separation of the data for ethanol from the data for acetone and 2-propanol.

Following these principal component analyses, the supervised data classification method of linear discriminant analysis (LDA) was performed on the rugate wavelength data. The wavelength shifts of the rugate reflectance band recorded during each solvent dosing period were combined into one matrix. The 40 columns corresponding to the steady state part of each of the dosing periods and the 10 columns corresponding to the first purge periods for each solvent were selected and combined into a new dataset. This combined matrix was transposed so that time was set as the observation variable and pixel positions were set as the properties to explain each observation. LDA was performed on this transposed dataset, and the first three linear discriminants were investigated.

**Figure 9:**
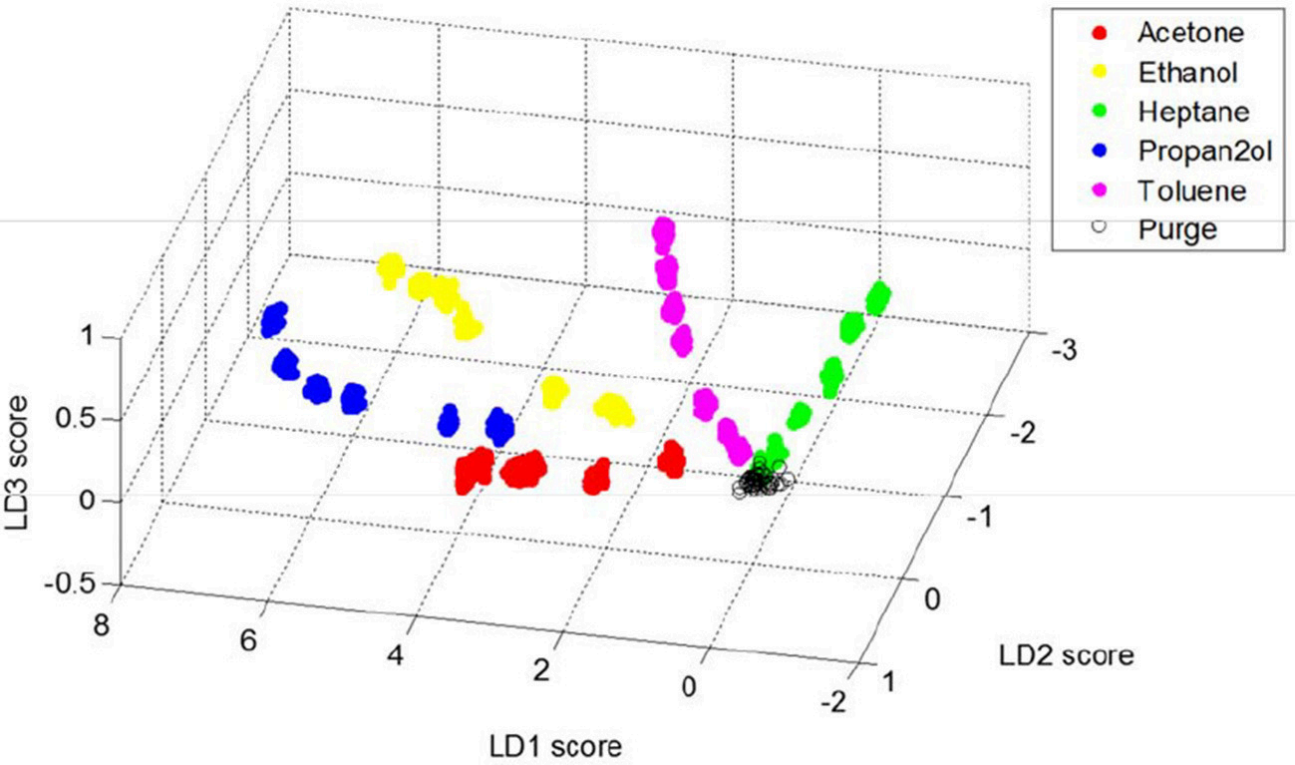
Plot showing the first three linear discriminant scores for rugate reflectance band wavelength shift data corresponding to steady-state dosing or purging of acetone, ethanol, heptane, 2-propanol and toluene.

A 3D plot with the scores from the first three linear discriminant axes was generated to capture the variation due to these components (Figure 9). The 3D plot has been rotated to optimize the visible separation between each solvent and each concentration. The plot shows separation due to both solvent identity and concentration, and all solvents are distinguished without any overlap. All the data for each solvent fall on a curve that starts near where the data representing the purge periods occur. The plot indicates that most of the variation in the data is explained by LD1 and LD2, however LD3 distinguishes acetone and ethanol from 2-propanol.

Principal component analysis on all the data, and principal component and linear discriminant analysis on just the data at the steady-state times of dosing and purging were also conducted on the hue data obtained from the color images. The loadings for PC1 of the complete dataset showed an abrupt change corresponding to the transition between the two pore-wall surface modifications, with the loadings being close to zero for the methylated side. The loadings for the oxidized side varied almost linearly from positive to negative, consistent with the observed change in direction of the hue change evident in the false color map in Figure 6. The scores plot for PC1 clearly showed the dosing for the oxygen-containing solvents acetone, ethanol, and 2-propanol with smaller responses for toluene and no responses for heptane. PC2 and PC3 did not contain chemically-interpretable variations. LDA was also unable to distinguish between the solvent vapors using the hue information.

## Discussion and Conclusions

Previous studies have investigated enhancing the selectivity of porous silicon sensors to detect and discriminate a wide range of vapors. Different solvent vapors were used in this study, and were easily grouped into hydrophilic and hydrophobic vapors. A previous study of porous silicon optical sensors had shown that 2-propanol vapor had stronger adsorption on hydrophilic surfaces, while toluene vapor had stronger adsorption on hydrophobic surfaces (Kelly et al., 2011a). A separate study with stacked layers of porous silicon showed that non-polar analytes (such as cyclohexane, heptane, and toluene) did not interact with the silica surface (hydrophilic) while polar molecules were strongly adsorbed. That study also noted that Knudsen diffusion was the primary mode of mass transport of the vapor within the pores of porous silicon (Kelly et al., 2011b). The results reported in this paper are consistent with observations of interactions of 2-propanol and heptane with thermally oxidized and methyl porous silicon by Ruminski et al. (2010).

An aim of the present research was to investigate whether porosity gradients can provide added selectivity for vapor sensing. Gao et al. (2002) had proposed that decreased pore sizes offered greater sensitivity, since smaller pores offer greater surface areas, therefore the optical response for a given vapor concentration should be higher. This was tested by creating porous silicon samples with a gradient in pore sizes across the sample during the fabrication step, using a thin wire counter electrode placed above the center of the exposed silicon wafer. This method delivers different current densities at different positions on the porous silicon sample, since the center of the silicon is closest to the counter electrode, therefore the current density at the center is larger than at the edges, and this current density gradient can cause a gradient in pore sizes porous silicon (Collins et al., 2002). The pore size gradient was not measured directly in this study, but the UV-Vis reflectance characterization experiments confirmed the gradient in rugate reflectance band wavelength across the porous silicon sample (Li et al., 2005). The present investigation showed that the optical and spectral changes of the porous silicon upon dosing did not correlate with the rugate reflectance band wavelength across the porous silicon surface. If the smaller pore sizes expected to be associated with the shorter wavelengths of the rugate reflectance band had increased the optical response of the porous silicon, and if the optical response change is captured by the camera, we would have expected greater sensitivity at the edges of the porous silicon sample and this was not observed. Moreover, the variations in sensitivity and selectivity that were observed across the porous silicon were more localized than if they had been caused by the current density gradient, showing that other factors were controlling the adsorption to a greater extent.

The response of the porous silicon sample was investigated using a color camera and a hyperspectral imager. Rugate filter porous silicon samples have been previously studied by monitoring the wavelength shift of the rugate peak using reflectance spectra measured at a single point on the surface (e.g., King et al., 2007; Ruminski et al., 2008). These and similar studies have shown that the wavelength of the rugate reflectance band shifts to the red upon solvent exposure and have also shown that the porous silicon sensor can selectively detect different vapors; however these papers only reported measurements at a single point (or several points) on the porous silicon sensor and not the whole sample.

As noted in the introduction, a previous study of the degradation of porous silicon used digital imaging combined with calculation of hue and a hue-related parameter (Ariza-Avidad et al., 2014). Those authors also noted that the apparent color of a rugate filter of wavelength lower than 560 nm was dependent on the white balance value of the color camera. This behavior is consistent with the observations in the current paper, where the change in hue upon dosing with solvent vapors had different signs depending on the initial hue or spectrum of the porous silicon at that position.

Finally, a recent study used color camera images to monitor the response of porous silicon under different concentrations of ethanol vapor (Park et al., 2010). That paper reported the color, current and photoluminescence responses of porous silicon as a function of ethanol vapor concentration. The authors reported that the color difference of the porous silicon was an efficient way of monitoring the optical response of the porous silicon. The color response of that study was observed under fluorescent lighting, whereas our study was conducted using broad-band white light. It is very likely that the sensitivity observed in the prior study is mainly due to the use of the fluorescent light source with its narrow band emissions in the red and green. The effect of different light sources on the information that can be obtained from photonic crystal sensors needs further study.

In the present research hyperspectral and RGB images of a transect across the whole porous silicon were captured during vapor dosing. These images were used to monitor the changes in reflectance of the porous silicon sample under as similar conditions as possible, so that these imaging techniques could be compared. The results showed that the hyperspectral imager was better at detecting small changes in optical response compared to RGB imaging for these porous silicon samples. In addition, it provided wavelength change information along a complete transect of the porous silicon sample, whereas the RGB images only showed measurable responses at some positions of the porous silicon samples. The capability of color images to collect changes in optical reflectance of porous silicon can be further explored by using variables other than hue.

There are many methods for the multivariate image analysis of time series such as images obtained during our dosing of porous silicon. However, there is no particular method to analyse image data that has been shown to be optimal for such situations in terms of sensitivity, selectivity, speed, and use of computer resources. In this study, two common multivariate statistical methods were used. One method (principal component analysis, PCA) was used for exploratory analysis, and the other method (linear discriminant analysis, LDA) was used in attempts to distinguish between already known classes (solvents and concentrations) and so further distinguish the groupings within the data. The results of the multivariate analysis showed that LDA provided better separation of solvent and dosing concentration classes using the hyperspectral imaging data than did PCA. The main reason that LDA did not work well for the color imaging data is that some dosing pulses resulted in no hue changes while the small changes meant that other dosing pulses had very similar hue changes. The discrimination was also adversely affected by there being little to no hue change on the methylated side of the OM porous silicon. This is likely to be due to the small shift in the spectral response, the initial wavelength of the rugate reflectance band, and the contribution from the interference fringes to the red of the rugate band.

## Author Contributions

SC conducted the experiments and data analysis and drafted part of the manuscript. GM designed the project, advised on the experiments and data analysis, and completed the manuscript.

### Conflict of Interest Statement

The authors declare that the research was conducted in the absence of any commercial or financial relationships that could be construed as a potential conflict of interest.
